# The association of metabolic syndrome with rotator cuff tendinopathy: a two-sample Mendelian randomization study

**DOI:** 10.1186/s13098-023-01189-5

**Published:** 2023-10-24

**Authors:** Ziqin Cao, Qiangxiang Li, Yajia Li, Jianhuang Wu

**Affiliations:** 1 Department of Spine Surgery and Orthopaedics, Xiangya Hospital, Central South University, Changsha, China; 2 National Clinical Research Center for Geriatric Disorders, Xiangya Hospital, Central South University, Changsha, China; 3 Ningxia Geriatric Disease Clinical Research Center, People’s Hospital of Ningxia Hui Autonomous Region, Hui Autonomous Region, Yinchuan, 750001 Ningxia China; 4 Department of Hunan Institute of Geriatrics, Hunan People’s Hospital, Changsha, China; 5 Department of Dermatology, Xiangya Hospital, Central South University, Changsha, 410011 Hunan China

**Keywords:** Metabolic syndrome, Rotator cuff tendinopathy, Body mass index, Waist circumference, Mendelian randomization

## Abstract

**Background:**

Observational research reported the underlying correlation of metabolic syndrome (MetS) and its components with rotator cuff tendinopathy (RCT), but their causality remained unclear. This study aimed to investigate whether genetically predicted MetS was related to the risk of RCT.

**Methods:**

Both univariable and multivariable Mendelian randomization (MR) analysis was applied using summary-level data from the most comprehensive genome-wide association studies to estimate the associations of MetS and its component with RCT, with the inverse variance weighted (IVW) as the primary method, and the method of Causal Analysis Using Summary Effect Estimates (CAUSE) as a supplement for false positives detection. The mediation analysis was furtherly used for the assessment of direct and indirect effects.

**Results:**

Univariable analysis revealed that genetically predicted MetS (OR: 1.0793; 95% CI 1.0311 to 1.1297), body mass index (BMI) (OR 1.2239; 95% CI 1.1357 to 1.3189), and waist circumference (WAC) (OR 1.3177; 95% CI 1.2015 to 1.4451) had a significant positive association with the risk of RCT. Triglycerides and systolic blood pressure were suggestively associated with RCT risk. These associations were also identified by CAUSE. There was independent causality of BMI (OR: 1.1806; 95% CI 1.0788 to 1.2920) and WAC (OR 1.3716; 95% CI 1.2076 to 1.5580) on RCT after adjustment for confounders. No mediator was found in the causal associations.

**Conclusion:**

Our study revealed the genetic causality of MetS and its components, especially BMI and WAC, with RCT risk. Early prevention and diagnosis of excess central adiposity contributing to MetS are significant in the RCT risk management.

**Supplementary Information:**

The online version contains supplementary material available at 10.1186/s13098-023-01189-5.

## Introduction

Shoulder pain ranks third among primary care musculoskeletal consultations and has a self-reported prevalence ranging from 16% to 26%[1–3]. Rotator cuff syndrome (RCS), also called rotator cuff tendinopathy (RCT) produces major clinical signs of pain and weakness during external rotation and elevation and is considered to account for over 70% of conditions of shoulder pain[2]. The condition involves subacromial structures, such as rotator cuff tendinitis/tendinosis, subacromial bursitis, and shoulder impingement syndrome[4–6]. RCT is commonly refractory to treatment, leading to restriction of daily activities and considerable socio-economic burden[7]. Extrinsic and inherent mechanisms associated with RCT are multifactorial and understanding of the condition is limited as a result.

Metabolic syndrome (MetS), is known as a complex group of metabolic disorders including central obesity, elevated blood pressure, hyperglycemia, and hyperlipidemia. The condition affects billions of the worldwide population and shows a rising global prevalence[8, 9], contributing to a serious health burden and mortality[10–12]. Epidemiological studies have indicated a potential relationship between MetS and RCT. Pooled meta-analyses have shown that diabetes [13] and dyslipidemia [14] are associated with a respective 1.24 and 1.17-fold increased risk of RCT. Hyperglycemia, hypertension, and excess body weight have also been found to be linked with symptomatic rotator cuff tears[15]. It has also been shown that hypertension [16] and excessive waist circumference [17] were associated with increased RCT risks in different observational studies. However, associations of MetS or its components with RCT identified by traditional observational studies are likely to be affected by confounders, limited sample capacity, short follow-up time, and reverse causation[18]. Thus, any causal effects of MetS in determining RCT risk remain unclear.

This study has applied Mendelian randomization (MR) analysis, an approach of causal inferences with relative robustness, to investigate associations of MetS with RCT to overcome the limitations in traditional study designs. Genetic variants closely related to exposure were used as instrumental variables (IVs) to eliminate the effects of confounders or reverse causality[18–20]. Genome-wide association studies (GWAS) have provided IVs associated with the target phenotype. The IVs associated with 10 predominant ingredients for MetS were identified, including body mass index (BMI), waist circumference (WAC), serum triglycerides (TC), high-density lipoprotein (HDL) cholesterol, fasting serum insulin (INS), fasting serum glucose (GLU), type 1 and type 2 diabetes (T1D and T2D), diastolic blood pressure (DBP), and systolic blood pressure (SBP). Additional mediators, such as alcohol and cigarette consumption, were also taken into consideration. A two-sample MR method, including both univariable MR (UVMR) and multivariable MR (MVMR), was used to explore genetic causal associations of MetS or its component conditions with RCT.

## Methods

### Data sources and instrumental variables selection

This study was guided by the Strengthening the Reporting of Observational Studies in Epidemiology using Mendelian Randomization (STROBE-MR) Statement.The following principal assumptions were made (Additional file 1: Figure S1): genetic variables should be strongly related to the exposure and variables could only have effects on the outcome through exposure with effects being independent of any confounding factors. Publicly available summary-level data was used and no additional ethical approval was needed. RCT summary data was obtained from the FinnGen Biobank Analysis Consortium database (Release 8, https://finngen.gitbook.io/documentation/) with diagnosis according to ICD-10 (International Classification of Diseases) criteria. A total of 274320 participants, including 21998 cases and 253122 controls, of European ancestry, were analyzed. The MetS dataset was obtained from a large GWAS study [21] using the UK biobank cohort, including 291107 participants aged 40–69 years of European ancestry. BMI (mg/kg^2^) data was obtained from a large GWAS meta-analysis which included 681275 individuals of European ancestry[22], WAC (462,166 individuals), HDL (403,943 individuals), and TC (441,016 individuals) were obtained from the UK biobank database[23]. Recent GWAS datasets for T1D (n=24840)[24] and T2D ( n= 655666)[25] patients of European ancestry were also used. GLU and INS were obtained from two European GWAS cohorts with 200622 and 151013 participants, respectively[26]. SBP and DBP were obtained from a European cohort of the International Consortium of Blood Pressure with 757601 individuals. MVMR and a two-step mediation MR model were constructed for the component conditions of MetS to adjust the mediation effect for potential confounders. Five mediators were adjusted for BMI, WAC, HDL, and TC, including alcohol intake, cigarette consumption, age at recruitment, genetic sex, and level of physical activity (moderate or vigorous). BMI was additionally adjusted as a mediator for the other six components of MetS. All mediators were obtained from different databases than the one used for the outcome to avoid the influence of sample overlap. A strict genome-wide significant threshold of *p* < 5 × 10^-8^ was set to filter out all strongly related SNPs and a process of the clump with a window of kb = 10000 and r^2^ = 0.001 was performed to avoid linkage disequilibrium (LD). The phenome-wide association studies database (pheWAS) was also matched to prevent the potential linkage between SNPs and confounders with the threshold of p-value < 5 × 10^-6^. All the estimates are given per one SD unit increase and the effect size is presented as the odds ratio (OR) with a 95% confidence interval (CI).

### Statistical approach

UVMR was performed using R packages MRPRESSO (version 1.0) and Two-Sample MR (version 0.5.6). Six different statistical methods were combined to evaluate the causal effects of MetS on RCT and a Bonferroni correction was applied to the UVMR to avoid multiple comparison errors. A post-correction p threshold < 0.0083 was considered statistically significant and 0.0083 < p < 0.05 was considered suggestively significant.

The inverse-variance weighted method (IVW) produces a relatively accurate assessment by combining all the Wald values of causality for each IV, with the assumption of invalid genetic instruments [27, 28]. The MR-Egger and MR pleiotropy residual sum and outlier (MR-PRESSO) methods were used to test the violation caused by directional pleiotropy. The intercept of the MR-Egger regression quantified pleiotropy across IVs, and the MR-Egger method could estimate a relatively robust value independent of IV validity, and obtain an adjusted result via the regression slope and intercept [29, 30]. MR-PRESSO was used for the detection of distorted effects related to horizontal pleiotropy and provided a corrected causal effect. The weighted model allowed the generation of a valid MR result even when the majority of IVs were invalid [31]. The weighted-median estimator method could give a consistent valid inference under the condition of over 50% of valid IVs [32]. MR-Robust Adjusted Profile Score (MRAPS) was applied to obtain an accurate estimate for the causality when there was ideally independent of IVs [33]. However, other methods produced wider confidence intervals (CI) than the IVW method and were only considered complements [34]. Thus, the MR-Egger Regression Model would be applied when significant pleiotropy was detected, and the MR-PRESSO model was applied to detect final outliers. Otherwise, the results by the IVW result method were given priority.

MVMR and two-step mediation MR were performed using the R packages, Two-Sample MR (version 0.5.6), MVMR (version 0.3.0), and MendelianRandomization (version 0.5.1). Random-effect IVW and MR-Egger models were constructed in MVMR and selected IVs were further analyzed by the two multi-variable models with an independent significance level of p < 0.05. MR-Egger results were prioritized under the condition of significant pleiotropy (p-value < 0.05). A two-step mediation MR analysis based on the difference method [35] was conducted to measure the mediation effects and mechanisms for each mediator. The total effect was estimated by the IVW method without correction of heterogeneity or pleiotropy and the direct effect was estimated using the multivariable IVW method. The indirect effect of MetS on RCT via the mediator was generated from the differences between total and direct estimates and the proportion mediated was calculated if the indirect effect was significant [36]. A value of p < 0.05 was considered statistically significant in the MVMR and mediation MR analysis.

### Sensitivity analysis

F-statistics were used to test the first assumption, calculated according to the formula: $F = \left( {\frac{n - k - 1}{k}} \right)\left( {\frac{R^2 }{{1 - R^2 }}} \right)$ (n: sample size; k: number of instrumental variables; R2: variance of exposure explained by selected instrumental variables generated by the MR Steiger directionality test). The conditional F-statistic was generated using the R package, MVMR, and was used to measure the instrument strength in MVMR to test whether the SNP strongly predicts each exposure conditional on the other exposures. Both an F or conditional F < 10 suggested significant weak instrument bias. Cochran’s Q-statistic was used to assess the heterogeneity caused by invalid IVs and a p-value < 0.05 was considered to indicate significant heterogeneity [38] in which case, a random-effect IVW model was adopted. The Causal Analysis Using the Summary Effect Estimates (CAUSE) method which utilizes the full genome-wide summary results rather than genome-wide significant loci only was used to correct the bias due to correlated and uncorrelated horizontal pleiotropy. Sample overlap was corrected by combining the largest sample sizes available for both exposure and outcome and improving the statistical power. Those associations inconsistent with the results of CAUSE were likely to have a false-positive error [39]. An arbitrary value of p < 1 × 10^−3^ for SNPs was set for the CAUSE analysis. If the pareto-k-diagnostic test reported a high-risk k value, a more strict p threshold would be adopted. A value of p < 0.05 was considered statistically significant for CAUSE analysis. The proportion of phenotypic variance explained by all SNPs (R2xy) was calculated by MR Steiger methods and was used to measure heritability. A binary-outcome-model from the mRnd tools was used to evaluate the statistical power of the current study (https://shiny.cnsgenomics.com/mRnd/). A power lower than 80% would be considered insufficient and the minimum sample size required at 80% power calculated. Leave-one-out analysis was performed to detect an unstable SNP which showed a disproportionately large individual influence on IVW regression coefficients. The identification of such an SNP indicates that conclusions should be drawn with caution[31].

## Results

### Genetic instruments

A total of 77 SNPs were identified as IVs for MetS, 475 were for BMI, 344 were for WAC, 308 were for HDL, 267 were for TC, 38 were for T1D, 113 were for T2D, 60 were for GLU, 37 were for INS, 426 were for SBP and 430 were for DBP (Additional file 2:Table S1-S11). The sensitivity analysis is shown in Table 1. MetS (11.96%), HDL (11.76%), TC (9.63%), T1D (17.93%), and T2D (13.95%) showed relatively high heritability but BMI (4.91%), WAC (4.49%), GLU (4.40%), INS (1.75%), SBP (4.63%) and DBP (4.80%) showed relatively low heritability. F-statistics indicated high instrumental strength for all exposures, ranging from 63.04 to 940.31. HDL (6%), T1D (7%), T2D (51%), GLU (43%), SBP (5%), and DBP (5%) all generated insufficient statistical power, perhaps due to insufficient sample size and low heritability.

**Table 1:** Sensitivity analysis of the UVMR

Exposure	Number of IVs	Heterogeneity test		MR-Egger pleiotropy test		MR-PRESSO global and distorted outlier test			R2xz	F statistics	Statistics power		
		Q value	P-value	Intercept	P-value	RSSobs	P-value	Number of outliers			Power	NCP	Minimum number of participants at 80% power
Metabolic syndrome	77	656.4696	5.13E-08	0.0021	2.16E-01	659.3605	3.00E-04	2	11.96%	75.6922	97%	14.53	NA
Body mass index	475	486.6747	5.04E-07	0.0000	9.83E-01	512.8289	2.00E-04	7	4.91%	73.9818	100%	46.67	NA
Waist circumference	344	466.5209	1.08E-08	−0.0029	3.28E-02	479.2033	2.00E-04	6	4.49%	63.0433	100%	84.94	NA
HDL cholesterol	308	336.4586	2.20E-03	0.0024	7.07E-02	363.4878	1.00E-04	7	11.76%	174.6032	6%	0.07	32,989,842
Triglycerides	267	44.7670	1.78E-01	−0.0033	3.88E-01	57.3322	1.04E-01	5	9.63%	175.9911	83%	8.46	NA
Type 1 diabetes	38	148.4480	1.20E-02	0.0072	1.85E-02	164.9014	5.00E-03	0	17.93%	142.5518	7%	0.16	NA
Type 2 diabetes	113	96.4514	1.51E-03	−0.0030	3.99E-01	108.7939	3.00E-04	2	13.95%	940.3147	51%	3.96	544,383
Fasting glucose	60	50.3129	5.70E-02	−0.0014	8.53E-01	52.8547	5.90E-02	3	4.40%	153.7989	43%	3.17	678,174
Fasting insulin	37	556.1044	1.85E-05	−0.0017	3.15E-01	603.9387	1.00E-04	0	1.75%	51.6940	84%	8.81	NA
Systolic blood pressure	426	614.9849	8.85E-09	−0.0014	3.99E-01	629.4504	1.00E-04	4	4.63%	86.3792	5%	0.02	113,387,310
Diastolic blood pressure	430	99.0027	3.94E-02	0.0053	1.25E-01	104.7258	4.87E-02	8	4.80%	88.7655	5%	0.02	1,599,783,962

### UVMR analysis

All traits showed significant heterogeneity except for INS (Q = 50.313; p = 0.057) while only T2D (intercept = 0.0072; p = 0.0185) and HDL (intercept =  0.003; p = 0.033) showed pleiotropy. No distorted effect outliers were detected by MR-PRESSO analysis.

**Fig. 1:**
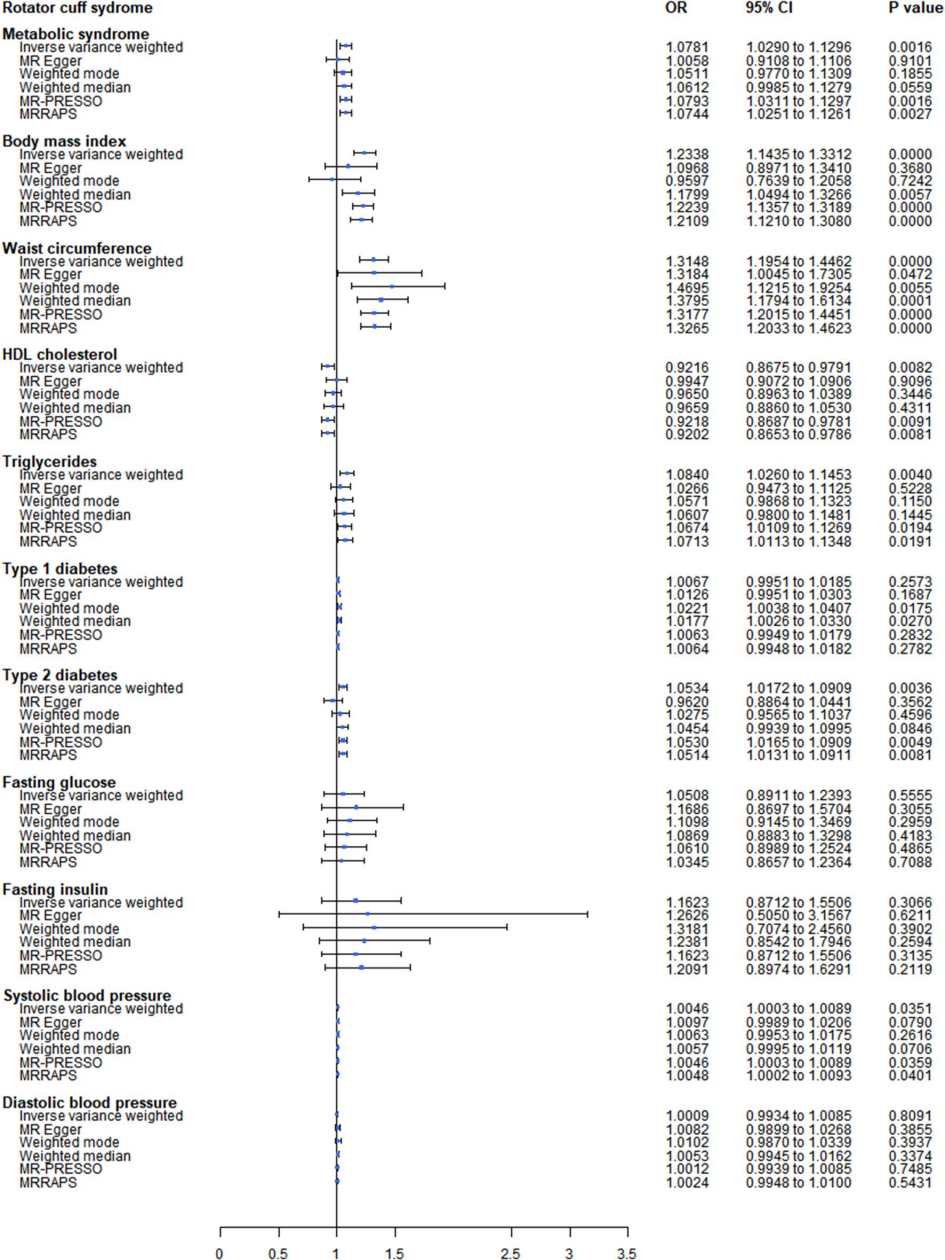
Forest plot of the univariable Mendelian randomization analyses exploring associations between childhood sunburn to skin carcinoma risk using different Mendelian randomization statistical models. OR: odds ratio; CIs: confidence intervals

MetS (OR: 1.0793; 95% CI 1.0311 to 1.1297; p = 1.57E-03), BMI (OR:1.2239; 95% CI 1.1357 to 1.3189; p = 1.82E-07) and WAC (OR: 1.3177; 95% CI 1.2015 to 1.4451; p = 1.05E-08) were all causally related to RCT while TC (OR 1.3177; 95% CI 1.2015 to 1.4451; p = 1.94E-02) and SBP (OR 1.0045; 95% CI 1.0003 to 1.0089; p = 3.59E-02) had a suggestively significant association with RCT. T2D also showed a significant positive effect on RCT (IVW OR: 1.10534; 95% CI 1.0172 to 1.0909; p = 3.57E-03). However, after the MR-Egger correction, no significant association between T2D and RCT remained (MR-Egger OR: 0.9620; 95% CI 0.8864 to 1.0441; p = 3.56E-01) (Fig. 1).

The CAUSE models gave similar results (Additional file 1:Figure S4). A genetically determined causal association was found for MetS-RCT (CAUSE OR 1.0408; 95% CI 1.0202 to 1.0725; p = 0.023), BMI-RCT (CAUSE OR 1.1503; 95% CI 1.0618 to 1.2461; p = 0.024) and WAC-RCT (CAUSE OR 1.2214; 95% CI 1.1504 to 1.2840; p = 0.001) and a suggestively significant association for TC (CAUSE OR: 1.0101; 95% CI 0.7558 to 1.3100; p = 0.859) and SBP (CAUSE OR: 1.0202; 95% CI 0.9900 to 1.0513; p = 0.520) were caused by horizontal pleiotropy.

The Wald ratio estimates for individual SNPs are described in Additional file 2: Table S12-S22. Leave-one-out plots are shown in Additional file 1: Figure S3 and indicated that no individual genetic variants appeared to significantly affect the results under the Bonferroni corrected threshold. Scatter plots are presented in Additional file 1: Figure S2.

### MVMR and mediation effect analysis

**Fig. 2:**
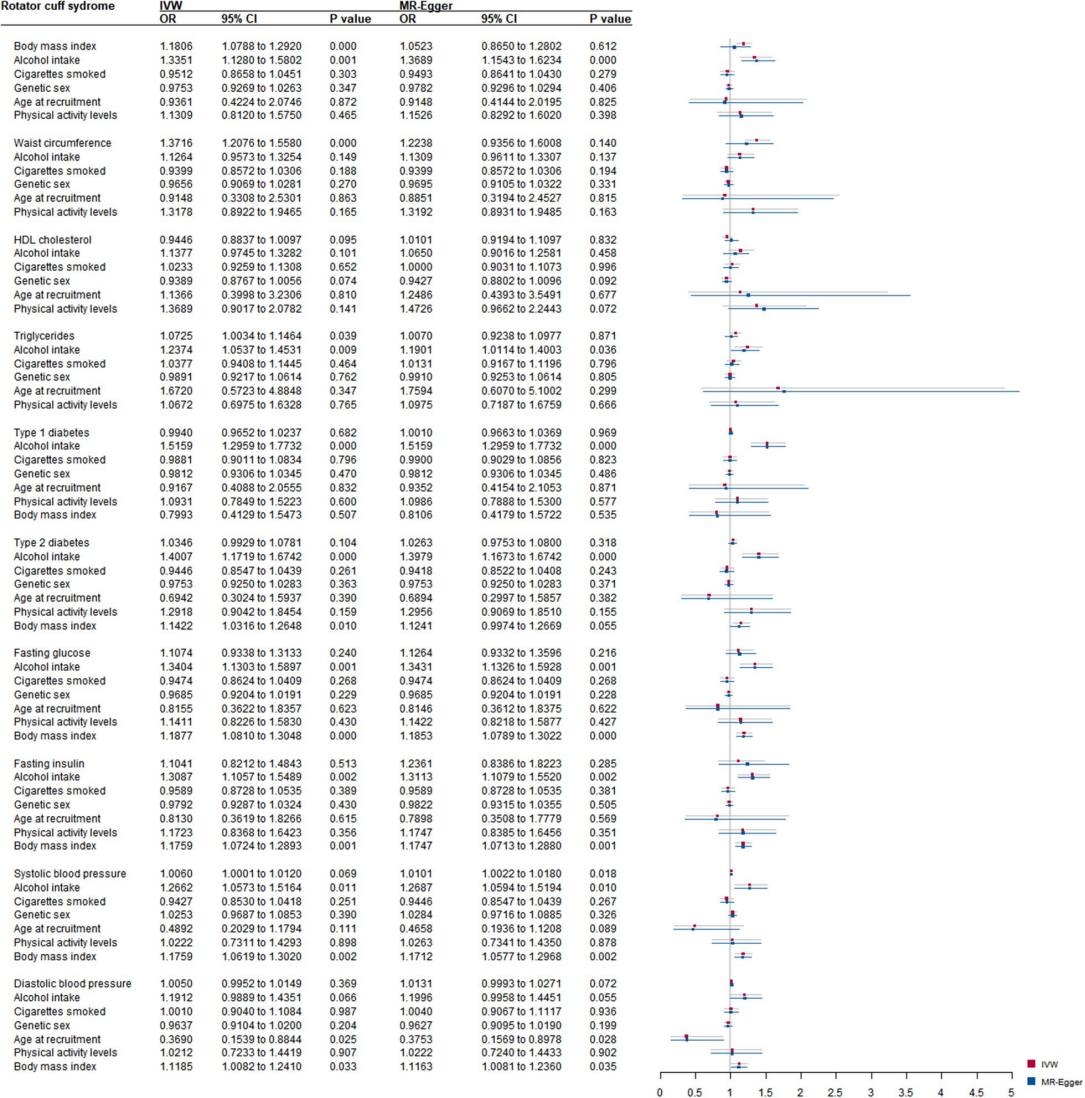
Forest plot of the multivariable Mendelian randomization analyses exploring genetically determined metabolic syndrome and its ingredients with rotator cuff syndrome risk-adjusted for confounding traits (alcohol intake, cigarettes consumption, age at recruitment, genetic sex, moderate to vigorous physical activity levels, and body mass index). OR: odds ratio; CIs: confidence intervals

MVMR models were built for MetS components to avoid the influence of other confounders (Fig. 2 and Additional file 2: Table S23). Weak instrumental strength was only found for T1D (conditional F = 2.5323) and INS (conditional F = 3.5613). Heterogeneity remained significant across all components.

Five mediators were controlled for BMI, WAC, HDL, and TC. Significant pleiotropy was only found in the MVMR models of HDL (intercept =  0.003, p = 0.045) and TC (intercept = 0.003, p = 0.024). There was strong evidence for a causal effect of BMI (OR: 1.1806; 95% CI 1.0788 to 1.2920; p = 0.000) and WAC (OR: 1.3716; 95% CI 1.2076 to 1.5580; p = 0.000) on the increased risk of RCT. T2D, GLU, INS, SBP, and DBP gave no significant pleiotropy in the MVMR models of T1D. No direct causal association was identified after controlling for six mediators.

**Fig. 3:**
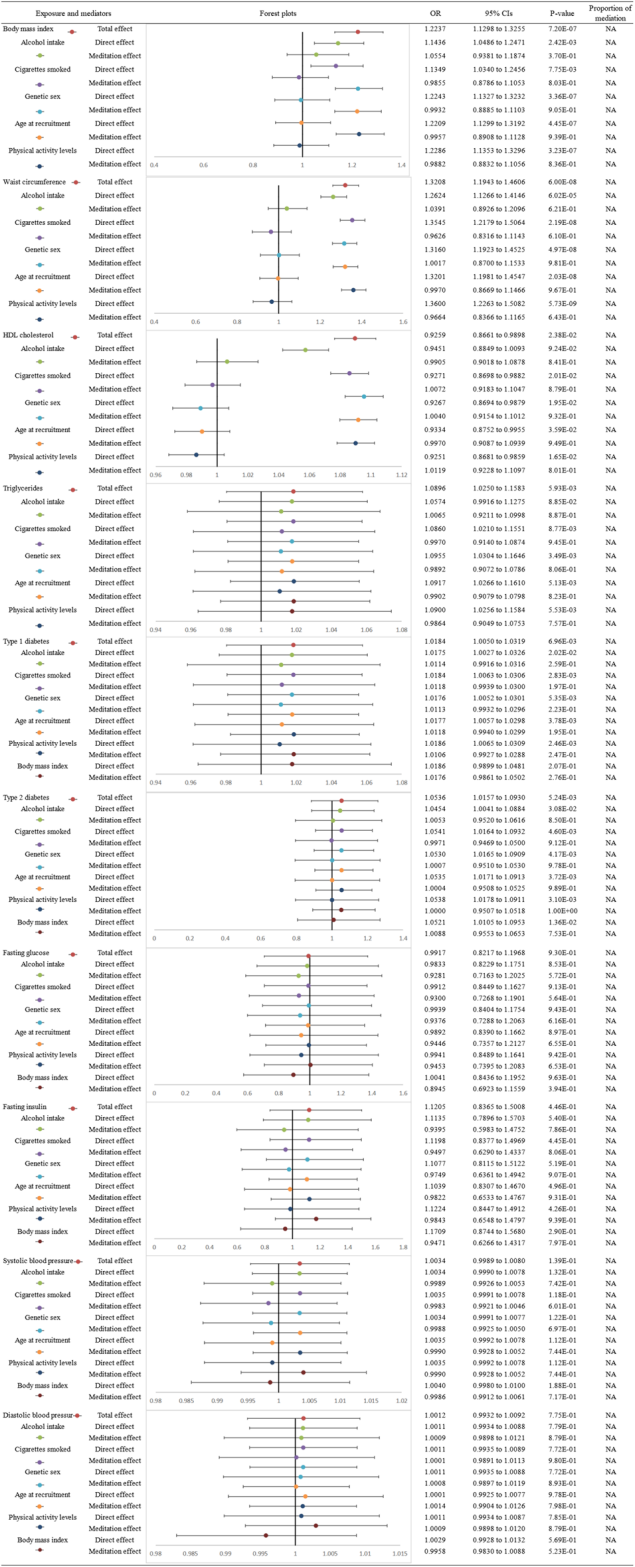
The forest plot of the mediation MR analyses. Causal estimates were given as odds ratio (OR) and 95% confidence intervals (CIs) for the effect of metabolic syndrome and its ingredients with rotator cuff syndrome risk. Red: total effect; Green: effect of alcohol intake; Purple: effect of cigarettes smoked; Blue: effect of genetic sex; Orange: effect of age at recruitment; Cyan: effect of physical activity levels; Brown: effect of body mass index

Details of mediation MR analysis are shown in Fig. 3. The directions of the total effect were mainly consistent with the UVMR results. BMI (total OR 1.2237; 95% CI 1.1298 to 1.3255; p = 7.20E-07), WAC (total OR 1.3208; 95% CI 1.1943 to 1.4606; p = 6.00E-08), HDL (total OR: 0.9259; 95% CI 0.8661 to 0.9898; p = 2.38E-02), TC (total OR: 1.090; 95% CI 1.0250 to 1.1583; p = 5.93E-03), T1D (total OR: 1.0184; 95% CI 1.0050 to 1.0319; p = 6.96E-03) and T2D (total OR: 1.0536; 95% CI 1.0157 to 1.0930; p = 5.24E-03) all had a significant effect on RCT occurrence. The direct effects of these components also remained significant while no significant indirect effect was identified. No mediator was thus found to influence the causal associations identified from UVMR.

## Discussion

UVMR during the current MR study revealed that genetically predicted MetS, BMI, and WAC had a significant positive association with the risk of RCT. TC and SBP were suggestively associated with RCT risk. The associations of MetS-RCT, BMI-RCT, and WAC-RCT were identified by CAUSE models. There was strong evidence for independent causal associations between both BMI and WAC and RCT after adjustment for confounders. No mediator was found in the causal associations identified from UVMR. Our study unveils a genetic predisposition to RCT in patients with MetS, and we hope that the insights gained will contribute to the enhancement of early diagnosis and monitoring of RCT risk in MetS populations. Given the rising adoption of sedentary lifestyles and physical inactivity, coupled with the increasing prevalence of metabolic syndrome with age, recognizing the heightened genetic risk of RCTs in patients with metabolic syndrome is crucial. This awareness is essential for ensuring appropriate early prevention and management of shoulder pain, especially among overweight MetS patients or those with excess WAC, in real-life medical practice. Additionally, these findings could suggest a subcategory of metabolic shoulder pain, thereby introducing new concepts for management and prognosis.

Epidemiological data have consistently suggested an association of RCT with cardiometabolic risk factors, such as obesity, BMI, and body fat [17, 31, 40–42]. Several meta-analyses have also indicated that blood glucose and body mass are potential risk factors for MetS, largely consistent with the current results[15]. However, previous observational design data for the impact of MetS on RCT risk have been inconclusive. Some MetS components, such as diabetes, hypertension, and dyslipidemia, have been proposed a significant positive association with RCT risk, [43, 44] but the possibility of bias existed for the three morbidities evaluated due to a lack of high-quality studies [45]. Obesity, hypertension, DM, and dyslipidemia are interrelated MetS components, making it hard to distinguish the contributions of the individual component versus the combination. The current MR analysis presents evidence supporting a determinate causal effect of some components of MetS, such as BMI and WAC on RCT. Suggestively significant associations found for TC and SBP may be false positives with observed associations being due to horizontal pleiotropy or confounders.

The underlying pathogenesis and mechanisms of RCT cannot be fully illustrated based on the available studies. Low-grade inflammatory biomarkers have been reported to be associated with RCT progression, although causality is unclear. MR analyses indicated that the genetically predicted risk of RCT is associated with high WAC (an indicator of central obesity) and BMI. Excess central adiposity has been considered a key component of MetS and adipose tissue may produce circulating proinflammatory cytokines (adipokines), such as tumor necrosis factor (TNF)-α, interleukin (IL)-1/6/1β, leptin and adiponectin. Elevated levels of cytokines IL-1 and IL-6 have been shown in the structure of subacromial bursa and partial thickness rotator cuff tear tissue [46–48]. Increased serum TNF-α has also been reported in the specimens of subacromial bursa extracted during symptomatic rotator cuff tendon surgery [49]. Decreased levels of the anti-inflammatory IL-10 have been found in obesity, insulin resistance, and dyslipidemia patients [50]. Increased proinflammatory and decreased anti-inflammatory adipokine expression may be a risk factor for RCT development [51]. However, these observations are speculative since no study has revealed appropriate biomarkers that might be possible regulatory factors between MetS and RCT. It should be noted that adipokines do not operate in isolation but interact with many other factors within a complex system.

The current MR study has the key strength of avoiding reverse causality and minimizing residual confounders. In addition, the summary-level dataset with predominant comprehensiveness and extensiveness for both RCT and MetS was utilized in this analysis, which provides high power in the causality investigation, as well as accurate estimated effect values. However, we also acknowledge some limitations. Firstly, the functions of the IVs and the pathway of their actual effects on the risk factors are not fully understood. Secondly, as weak instrumental strength was still found in T1D and INS in the MVMR results, these results should be interpreted with caution. Third, it is acknowledged that the pleiotropy effect could not be eliminated and may be obscured by potential confounders. Although we have adjusted for several significant common confounders in the MVMR and mediation MR models, considering the complex clinical and genetic backgrounds of both RCT and MetS, achieving complete avoidance of pleiotropy in MR analysis is nearly impossible. While significant covariates like occupational activity and concomitant tendinopathy could potentially influence the results, the absence of available high-quality datasets for these specific phenotypes currently hinders the verification of their potential pleiotropy. However, we believe that the bias in our results caused by pleiotropy is minimal, as several robust MR methods were employed. These methods can yield reliable inferences even if some genetic variants violate the IV assumptions. Last but not least, to mitigate biases stemming from population stratification and sample overlap, we strategically selected datasets from the FinnGen and UKB databases, confining our study to participants of European ancestry for exposure and outcomes, respectively. As a result, the findings of this study should be generalized to other populations with caution. Future investigations will necessitate the inclusion of high-quality GWAS datasets from diverse racial groups to determine the validity of our conclusions across varied genetic backgrounds. Furthermore, the absence of raw individual-level data limits our ability to conduct further stratified analysis on population subgroups.

## Conclusion

In conclusion, the current MR study revealed the genetic causality of MetS and its components, especially BMI and WAC, with the risk of RCT. The current findings indicate that the prevention, management, and treatment of RCTs need to be strengthened in a clinical setting by controlling the excess central adiposity that contributes to MetS.

### Supplementary Information

**Additional file 1: Figure S1. **Diagram for key assumptions of MR analyses.MR study relies on three assumptions: (I) the instrumental variables (IVs) should be associated with the exposure (MetS). (II) the IVs should not be related to confounders. (III) the lVs should influence the outcome (RCT) risk via the exposure, not through other pathways. Line with arrows indicate that the genetic instruments (SNPs) are associated with the exposure and could only affect the outcome via the exposure. Dashed lines indicate that the genetic instruments (SNPs) are independent of any confounding variables between the results. MR: mendelian randomization; MetS:metabolic syndrome; RCT: rotator cuff tendinopathy. **Figure S2.** Scatter plots of the univariable mendelian randomisation analyses. The slope of each line corresponding to the estimated MR effect in different models, including the conventional IVW, WM, WMM, MR-Egger, MR-RAPS and MR-PRESSO methods. The effect of A: MetS on RCT; B: BMI on RCT; C: WAC on RCT; D: HDL on RCT; E: TC on RCT; F: T1D on RCT; G: T2D on RCT; H: GLU on RCT; I: INS on RCT; J: SBP on RCT; K: DBP on RCT. BMI: body mass index; WAC: waist circumference; HDL: serum HDL cholesterol; TC: triglycerides; T1D: type 1 diabetes; T2D: type 2 diabetes; GLU: fasting serum glucose; INS: fasting serum insulin; SBP: systolic blood pressure; DBP: diastolic blood pressure. **Figure S3. **Leave-one-out stability tests of the univariable mendelian randomisation analyses. Calculate the MR results of the remaining IVs after removing the IVs one by one. The effect of A: MetS on RCT; B: BMI on RCT; C: WAC on RCT; D: HDL on RCT; E: TC on RCT; F: T1D on RCT; G: T2D on RCT; H: GLU on RCT; I: INS on RCT; J: SBP on RCT; K: DBP on RCT. BMI: body mass index; WAC: waist circumference; HDL: serum HDL cholesterol; TC: triglycerides; T1D: type 1 diabetes; T2D: type 2 diabetes; GLU: fasting serum glucose; INS: fasting serum insulin; SBP: systolic blood pressure; DBP: diastolic blood pressure. **Figure S4. **The forest plot of the CAUSE method MR analysis. Causal estimates were given as beta and 95% confidence intervals (CIs).

**Additional file 2. Table S1.** Information of identified SNPs in exposure (metabolic syndrome) and outcomes(rotator cuff syndrome). **Table S2.** Information of identified SNPs in exposure (body mass index) and outcomes(rotator cuff syndrome). **Table S3.** Information of identified SNPs in exposure (waist circumference) and outcomes(rotator cuff syndrome). **Table S4.** Information of identified SNPs in exposure (HDL cholesterol) and outcomes(rotator cuff syndrome). **Table S5.** Information of identified SNPs in exposure (triglycerides) and outcomes(rotator cuff syndrome). **Table S6.** Information of identified SNPs in exposure (type 1 diabetes) and outcomes(rotator cuff syndrome). **Table S7.** Information of identified SNPs in exposure (type 2 diabetes) and outcomes(rotator cuff syndrome).**Table S8.** Information of identified SNPs in exposure (fasting glucose) and outcomes(rotator cuff syndrome). **Table S9.** Information of identified SNPs in exposure (fasting insulin) and outcomes(rotator cuff syndrome). **Table S10.** Information of identified SNPs in exposure (systolic blood pressure) and outcomes(rotator cuff syndrome). **Table S11.** Information of identified SNPs in exposure (diastolic blood pressure) and outcomes(rotator cuff syndrome). **Table S12.** Wald ratio estimate results of individual SNPs in exposure (metabolic syndrome) and outcomes(rotator cuff syndrome). **Table S13.** Wald ratio estimate results of individual SNPs in exposure (body mass index) and outcomes(rotator cuff syndrome). **Table S14.** Wald ratio estimate results of individual SNPs in exposure (waist circum) and outcomes(rotator cuff syndrome). **Table S15.** Wald ratio estimate results of individual SNPs in exposure (HDL cholesterol) and outcomes(rotator cuff syndrome). **Table S16.** Wald ratio estimate results of individual SNPs in exposure (triglycerides) and outcomes(rotator cuff syndrome). **Table S17.** Wald ratio estimate results of individual SNPs in exposure (type 1 diabetess) and outcomes(rotator cuff syndrome). **Table S18.** Wald ratio estimate results of individual SNPs in exposure (type 2 diabetes) and outcomes(rotator cuff syndrome). **Table S19.** Wald ratio estimate results of individual SNPs in exposure (fasting glucose) and outcomes(rotator cuff syndrome). **Table S20.** Wald ratio estimate results of individual SNPs in exposure (fasting insulin) and outcomes(rotator cuff syndrome). **Table S21.** Wald ratio estimate results of individual SNPs in exposure (systolic blood pressure) and outcomes(rotator cuff syndrome). **Table S22.** Wald ratio estimate results of individual SNPs in exposure (diastolic blood pressure) and outcomes(rotator cuff syndrome). **Table S23.** Detailed result of the MVMR analysis.

## Data Availability

The data that support the findings of this study are available from the corresponding author upon reasonable request.

## Abbreviations

RCS: Rotator cuff syndrome
RCT: Rotator cuff tendinopathy
Mets: Metabolic syndrome
MR: Mendelian randomization
IVs: Instrumental variables
GWAS: Genome-wide association studies
WAC: Body mass index (BMI), waist circumference
TC: Triglycerides
HDL: High-density lipoprotein
INS: Insulin
GLU: Fasting serum glucose
T1D: Type 1 diabetes
T2D: Type 2 diabetes
DBP: Diastolic blood pressure
SBP: Systolic blood pressure
UVMR: Univariable MR
MVMR: Multivariable MR
STROBE-MR: Strengthening the Reporting of Observational Studies in Epidemiology using Mendelian Randomization
pheWAS: Phenome-wide association studies database
IVW: Inverse-variance weighted method
MR-PRESSO: MR pleiotropy residual sum and outlier
MRAPS: MR-Robust Adjusted Profile Score
CI: Confidence intervals
CAUSE: Causal Analysis Using the Summary Effect Estimates
TNF: Tumor necrosis factor
IL: Interleukin
