# Supplementary material for: The association of metabolic syndrome with rotator cuff tendinopathy: a two-sample Mendelian randomization study
Source: Diabetol Metab Syndr. 2023 Oct 24;15:211. doi: 10.1186/s13098-023-01189-5 (PMC10594889; doi:10.1186/s13098-023-01189-5)
Supplement: Supplementary file 1 — Additional file 1: Figure S1. Diagram for key assumptions of MR analyses.MR study relies on three assumptions: (I) the instrumental variables (IVs) should be associated with the exposure (MetS). (II) the IVs should not be related to confounders. (III) the lVs should influence the outcome (RCT) risk via the exposure, not through other pathways. Line with arrows indicate that the genetic instruments (SNPs) are associated with the exposure and could only affect the outcome via the exposure. Dashed lines indicate that the genetic instruments (SNPs) are independent of any confounding variables between the results. MR: mendelian randomization; MetS:metabolic syndrome; RCT: rotator cuff tendinopathy. Figure S2. Scatter plots of the univariable mendelian randomisation analyses. The slope of each line corresponding to the estimated MR effect in different models, including the conventional IVW, WM, WMM, MR-Egger, MR-RAPS and MR-PRESSO methods. The effect of A: MetS on RCT; B: BMI on RCT; C: WAC on RCT; D: HDL on RCT; E: TC on RCT; F: T1D on RCT; G: T2D on RCT; H: GLU on RCT; I: INS on RCT; J: SBP on RCT; K: DBP on RCT. BMI: body mass index; WAC: waist circumference; HDL: serum HDL cholesterol; TC: triglycerides; T1D: type 1 diabetes; T2D: type 2 diabetes; GLU: fasting serum glucose; INS: fasting serum insulin; SBP: systolic blood pressure; DBP: diastolic blood pressure. Figure S3. Leave-one-out stability tests of the univariable mendelian randomisation analyses. Calculate the MR results of the remaining IVs after removing the IVs one by one. The effect of A: MetS on RCT; B: BMI on RCT; C: WAC on RCT; D: HDL on RCT; E: TC on RCT; F: T1D on RCT; G: T2D on RCT; H: GLU on RCT; I: INS on RCT; J: SBP on RCT; K: DBP on RCT. BMI: body mass index; WAC: waist circumference; HDL: serum HDL cholesterol; TC: triglycerides; T1D: type 1 diabetes; T2D: type 2 diabetes; GLU: fasting serum glucose; INS: fasting serum insulin; SBP: systolic blood pressure; DBP: diastolic blood pressure. Figure [file 13098_2023_1189_MOESM1_ESM.docx]

**Additional file 1:Figure S1.** Diagram for key assumptions of MR analyses.MR study relies on three assumptions: (I) the instrumental variables (IVs) should be associated with the exposure (MetS). (II) the IVs should not be related to confounders. (III) the lVs should influence the outcome (RCT) risk via the exposure, not through other pathways. Line with arrows indicate that the genetic instruments (SNPs) are associated with the exposure and could only affect the outcome via the exposure. Dashed lines indicate that the genetic instruments (SNPs) are independent of any confounding variables between the results. MR: mendelian randomization; MetS:metabolic syndrome; RCT: rotator cuff tendinopathy.

**Additional file 1: Figure S2.** Scatter plots of the univariable mendelian randomisation analyses. The slope of each line corresponding to the estimated MR effect in different models, including the conventional IVW, WM, WMM, MR-Egger, MR-RAPS and MR-PRESSO methods. The effect of A: MetS on RCT; B: BMI on RCT; C: WAC on RCT; D: HDL on RCT; E: TC on RCT; F: T1D on RCT; G: T2D on RCT; H: GLU on RCT; I: INS on RCT; J: SBP on RCT; K: DBP on RCT. BMI: body mass index; WAC: waist circumference; HDL: serum HDL cholesterol; TC: triglycerides; T1D: type 1 diabetes; T2D: type 2 diabetes; GLU: fasting serum glucose; INS: fasting serum insulin; SBP: systolic blood pressure; DBP: diastolic blood pressure.

**Additional file 1: Figure S3.** Leave-one-out stability tests of the univariable mendelian randomisation analyses. Calculate the MR results of the remaining IVs after removing the IVs one by one. The effect of A: MetS on RCT; B: BMI on RCT; C: WAC on RCT; D: HDL on RCT; E: TC on RCT; F: T1D on RCT; G: T2D on RCT; H: GLU on RCT; I: INS on RCT; J: SBP on RCT; K: DBP on RCT. BMI: body mass index; WAC: waist circumference; HDL: serum HDL cholesterol; TC: triglycerides; T1D: type 1 diabetes; T2D: type 2 diabetes; GLU: fasting serum glucose; INS: fasting serum insulin; SBP: systolic blood pressure; DBP: diastolic blood pressure.

**Additional file 1: Figure S4.** The forest plot of the CAUSE method MR analysis. Causal estimates were given as beta and 95% confidence intervals (CIs).

**
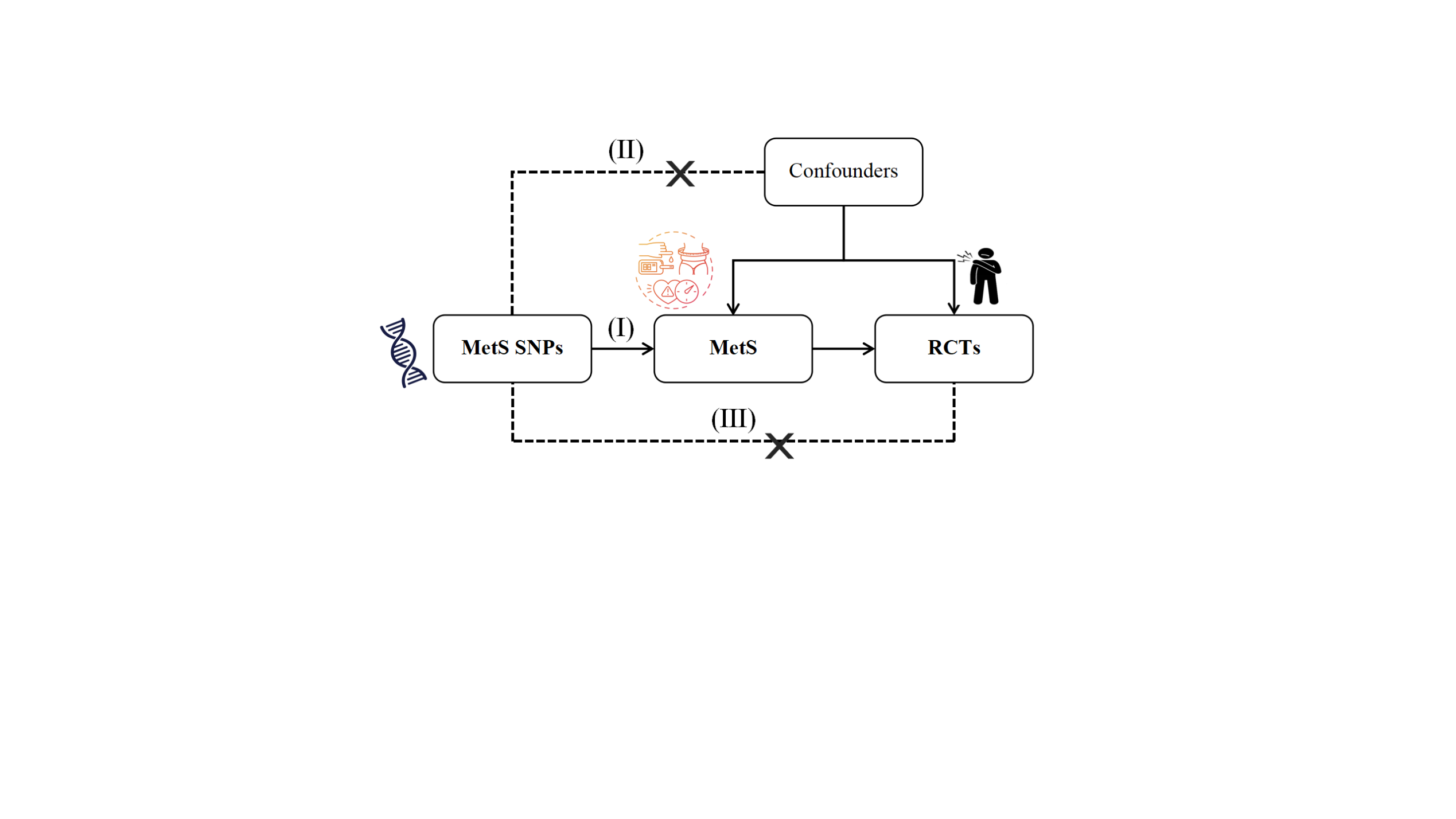
**

**Additional file 1: Figure S1.** Diagram for key assumptions of MR analyses.MR study relies on three assumptions: (I) the instrumental variables (IVs) should be associated with the exposure (MetS). (II) the IVs should not be related to confounders. (III) the lVs should influence the outcome (RCT) risk via the exposure, not through other pathways. Line with arrows indicate that the genetic instruments (SNPs) are associated with the exposure and could only affect the outcome via the exposure. Dashed lines indicate that the genetic instruments (SNPs) are independent of any confounding variables between the results. MR: mendelian randomization; MetS:metabolic syndrome; RCT: rotator cuff tendinopathy.


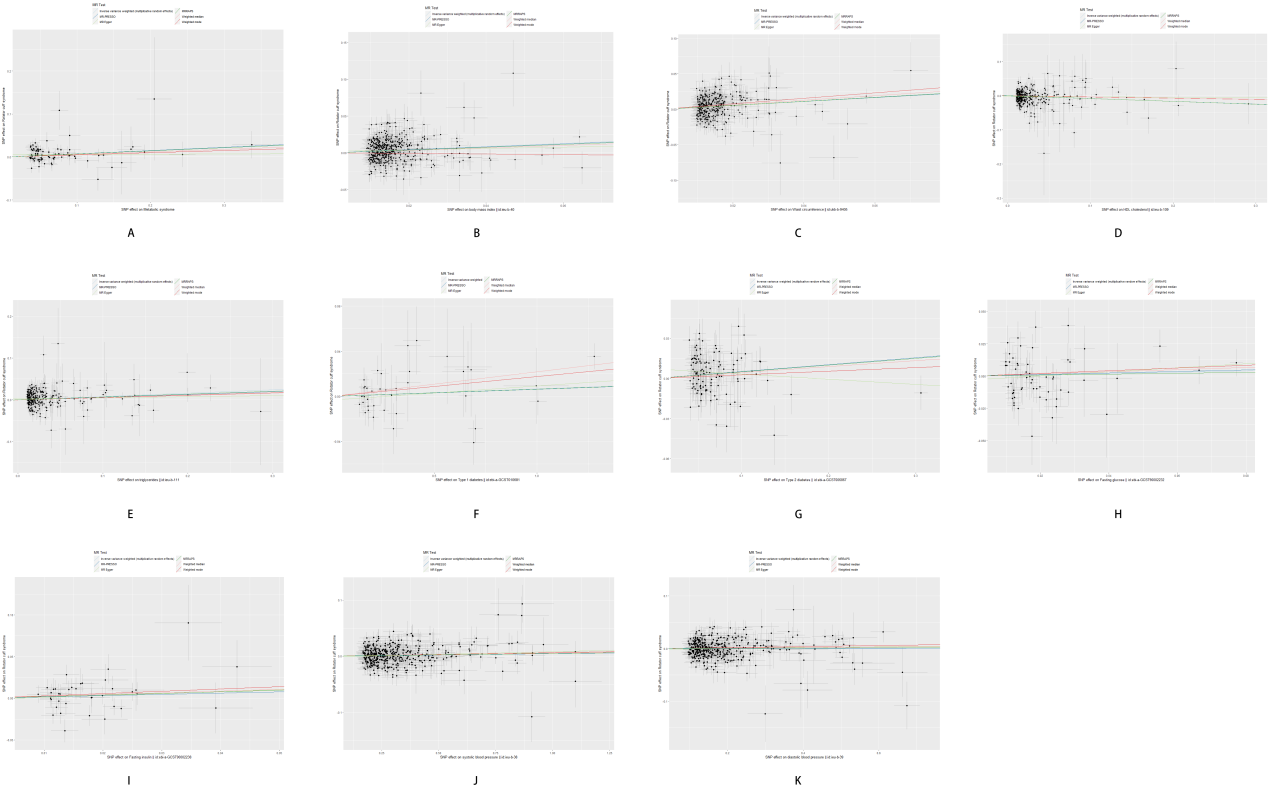


**Additional file 1: Figure S2.** Scatter plots of the univariable mendelian randomisation analyses. The slope of each line corresponding to the estimated MR effect in different models, including the conventional IVW, WM, WMM, MR-Egger, MR-RAPS and MR-PRESSO methods. The effect of A: MetS on RCT; B: BMI on RCT; C: WAC on RCT; D: HDL on RCT; E: TC on RCT; F: T1D on RCT; G: T2D on RCT; H: GLU on RCT; I: INS on RCT; J: SBP on RCT; K: DBP on RCT. BMI: body mass index; WAC: waist circumference; HDL: serum HDL cholesterol; TC: triglycerides; T1D: type 1 diabetes; T2D: type 2 diabetes; GLU: fasting serum glucose; INS: fasting serum insulin; SBP: systolic blood pressure; DBP: diastolic blood pressure.


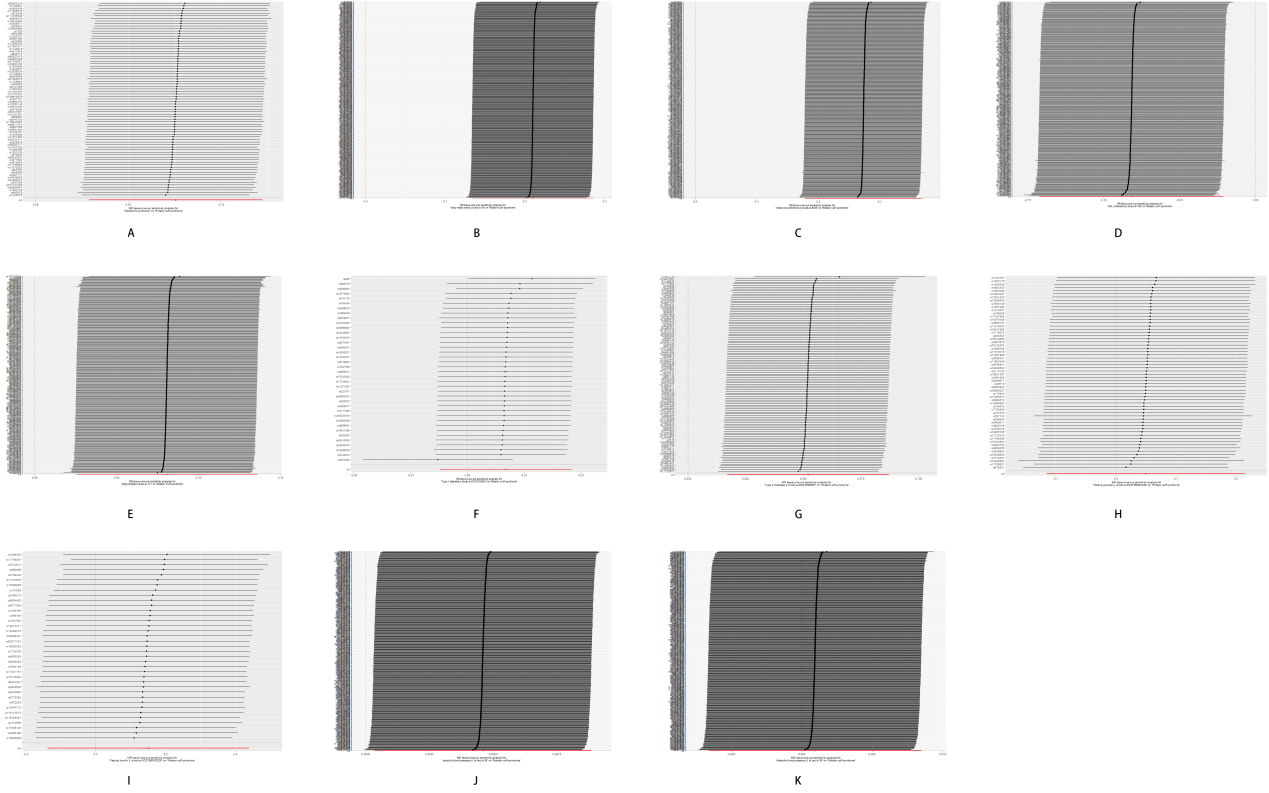


**Additional file 1: Figure S3.** Leave-one-out stability tests of the univariable mendelian randomisation analyses. Calculate the MR results of the remaining IVs after removing the IVs one by one. The effect of A: MetS on RCT; B: BMI on RCT; C: WAC on RCT; D: HDL on RCT; E: TC on RCT; F: T1D on RCT; G: T2D on RCT; H: GLU on RCT; I: INS on RCT; J: SBP on RCT; K: DBP on RCT. BMI: body mass index; WAC: waist circumference; HDL: serum HDL cholesterol; TC: triglycerides; T1D: type 1 diabetes; T2D: type 2 diabetes; GLU: fasting serum glucose; INS: fasting serum insulin; SBP: systolic blood pressure; DBP: diastolic blood pressure.


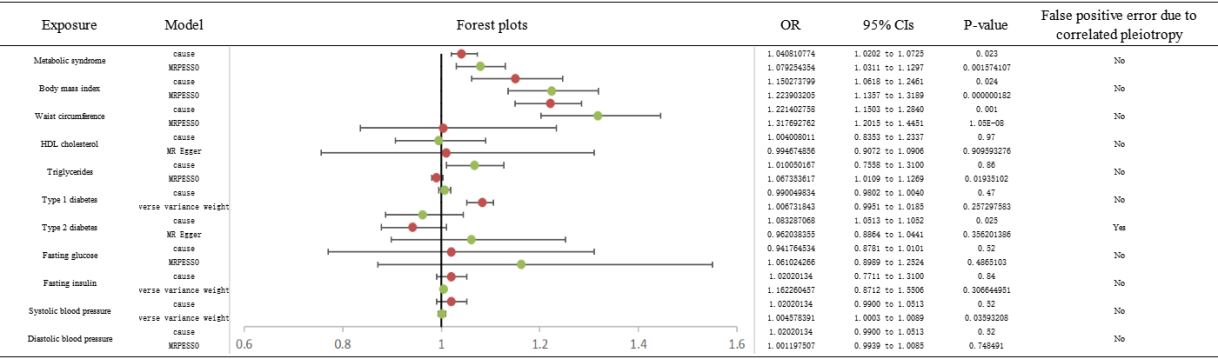


**Additional file 1: Figure S4.** The forest plot of the CAUSE method MR analysis. Causal estimates were given as beta and 95% confidence intervals (CIs).
